# Mortality during a Large-Scale Heat Wave by Place, Demographic Group, Internal and External Causes of Death, and Building Climate Zone

**DOI:** 10.3390/ijerph13030299

**Published:** 2016-03-09

**Authors:** Lauren Joe, Sumi Hoshiko, Dina Dobraca, Rebecca Jackson, Svetlana Smorodinsky, Daniel Smith, Martha Harnly

**Affiliations:** 1 Environmental Health Investigations Branch, California Department of Public Health, Richmond, CA 94804, USA; sumi.hoshiko@cdph.ca.gov (S.H.); dina.dobraca@cdph.ca.gov (D.D.); daniel.smith@cdph.ca.gov (D.S.); marthaharn@gmail.com (M.H.); 2 Occupational Health Branch, California Department of Public Health, Richmond, CA 94804, USA; rebecca.jackson@cdph.ca.gov (R.J.); svetlana.smorodinsky@cdph.ca.gov (S.S.)

**Keywords:** heat wave, mortality, relative risk, excess deaths, vulnerabilities, at-home, external cause of death, internal cause of death, building climate zone

## Abstract

Mortality increases during periods of elevated heat. Identification of vulnerable subgroups by demographics, causes of death, and geographic regions, including deaths occurring at home, is needed to inform public health prevention efforts. We calculated mortality relative risks (RRs) and excess deaths associated with a large-scale California heat wave in 2006, comparing deaths during the heat wave with reference days. For total (all-place) and at-home mortality, we examined risks by demographic factors, internal and external causes of death, and building climate zones. During the heat wave, 582 excess deaths occurred, a 5% increase over expected (RR = 1.05, 95% confidence interval (CI) 1.03–1.08). Sixty-six percent of excess deaths were at home (RR = 1.12, CI 1.07–1.16). Total mortality risk was higher among those aged 35–44 years than ≥65, and among Hispanics than whites. Deaths from external causes increased more sharply (RR = 1.18, CI 1.10–1.27) than from internal causes (RR = 1.04, CI 1.02–1.07). Geographically, risk varied by building climate zone; the highest risks of at-home death occurred in the northernmost coastal zone (RR = 1.58, CI 1.01–2.48) and the southernmost zone of California’s Central Valley (RR = 1.43, CI 1.21–1.68). Heat wave mortality risk varied across subpopulations, and some patterns of vulnerability differed from those previously identified. Public health efforts should also address at-home mortality, non-elderly adults, external causes, and at-risk geographic regions.

## 1. Introduction

Heat waves are projected to increase in frequency and duration, in California and globally [1,2]. Numerous studies have linked increases in mortality to heat wave exposures. A broad range of medical, social, and environmental public health interventions have been recommended internationally for the general public and subpopulations thought to be at increased risk. Current guidance highlights caution for young children, the elderly, persons with certain chronic conditions, and workers with outdoor or exertional exposures [3,4,5]. Climate adaptation and mitigation initiatives advocate for changes in urban design, more energy-efficient buildings, and education and outreach to vulnerable populations [5,6,7]. To inform these initiatives, researchers and public health agencies have called for additional identification of vulnerable populations, including local and regional vulnerability assessments [5,6,8]. Such efforts would be further enhanced by more detailed evaluations of subgroups within contextual factors, such as place of death, which would allow more specific risk characterization.

Place of death can provide a critical context for risk of death from heat. Those at home during heat events, rather than in an institution such as a hospital or nursing home, may be at greater risk due to fewer protective factors, including air conditioning (AC), medical care, and social support [9]. Mortality is higher outside of a hospital than inside a hospital in conditions of extreme heat [10]. However, with the exception of two studies from Canada [11,12], heat mortality studies based on death certificates have not specifically analyzed risks for people who were at home at the time of death. Housing characteristics, which may vary by geographic location and neighborhood, may also modify this risk [13].

Studies of demographic characteristics have identified women, blacks, the elderly, and the very young as populations particularly vulnerable to heat, although lack of consistency across studies has been seen as well [8,13,14,15]. Risks associated with these personal characteristics may be due to many factors including thermoregulatory capacity associated with aging or underlying medical conditions, access to medical care, level of physical activity, and the availability of AC. Few studies have examined heat-related mortality across detailed age categories or among other races/ethnicities including Hispanics or Asian/Hawaiian/Pacific Islanders (APIs), both significant populations in California [8,16]. Among the studies that have examined impacts by race, blacks have been frequently identified as more vulnerable, but many studies only evaluated black (or non-white) compared to white [8,13]. In a study of high ambient temperatures, Hispanics in California appeared less impacted than blacks or whites [16]. We did not locate any studies addressing heat-mortality risk among Asian Americans. 

Epidemiological studies of heat wave mortality impacts have typically used internal causes of death (*i.e.*, deaths due to natural causes not attributable to external factors) for analysis, and excluded external causes of death (*i.e.*, externally inflicted injuries or poisonings) [14,15,17,18,19,20,21,22,23,24,25,26,27]. While deaths specifically coded as due to extreme heat, classified as an external cause of death, have also been associated with elevated temperatures [12,28,29], other external causes of death (*i.e.*, transportation accidents, falls, accidental poisonings, drownings, homicides, and suicides) have only occasionally been examined in epidemiological studies of heat [30,31]. Symptoms of heat exhaustion, including confusion, irritability, and loss of coordination [32] may contribute to external causes of death not specifically ascribed to excessive heat.

The California heat wave that occurred in July 2006 was the longest and most widespread heat wave in the Western U.S. in 60 years [33]. The magnitude of the heat wave, and its occurrence in the ethnically and geographically diverse population of California, provides an opportunity to examine detailed subpopulation and contextual vulnerabilities. Previous analyses of this heat wave documented statewide increases in all-cause morbidity and mortality, but demographic differences in risk across the state were not examined [34,35]. Geographic analysis of several California counties found substantial differences, with a far greater mortality increase per temperature rise in a desert county than the urban, coastal county of Los Angeles [20].

Here, we examine the California 2006 heat wave to identify potentially vulnerable and resilient subpopulations by first evaluating total mortality by place of death, and then total and at-home mortality by detailed demographic group, internal and external causes of death, and California’s 16 building climate zones. The results of these analyses can assist local, regional, and global efforts to reduce heat-related health impacts.

## 2. Materials and Methods 

### 2.1. Mortality Data Source

Records of all deaths for June through August 2006 were obtained from the Center for Health Statistics and Informatics, California Department of Public Health (CDPH) [35]. International Classification of Diseases, Tenth Revision (ICD-10) diagnosis codes for underlying cause of death [36], place of death, residential address, and all demographic subgroup categories were obtained from the records. This study was conducted in accordance with the Declaration of Helsinki and the protocol was approved by the Committee for the Protection of Human Subjects of the California Health and Human Services Agency (protocol identification number: 12-04-0083).

### 2.2. Definitions of Heat Wave Deaths and Expected Deaths

The heat wave period in this study was an 18-day period between 15 July and 1 August, 2006. This included a 12-day “heat wave” defined by a previous meteorological analysis that found an exceptionally strong high pressure zone that created recorded-breaking temperatures [37], and an additional six days following the “heat wave” to allow for the inclusion of delayed or lagged impacts, as was done in previous analyses of this 2006 heat wave in California [14,34,35]. We defined a heat wave death as any death occurring during this 18-day heat wave period.

The reference period was drawn from the same summer (1 to 30 June, 6 to 14 July, and 8 to 31 August, 2006), which excluded a holiday period (1 to 5 July), the heat wave period, and the week immediately after the heat wave period. To calculate an expected number of deaths, we randomly selected 36 reference days from this reference period using a random number generator, matching two reference days to each heat wave period day by day-of-the-week to increase statistical precision. The number of reference days before and after the heat wave period was approximately the same.

### 2.3. Statistical Analysis: Relative Risk and Excess Deaths 

Similar to past studies, we used a simplified relative risk (RR) approach, comparing deaths occurring during a heat period to a reference period [34,35]. This approach assumes that the population size remains constant over the time of the study, an assumption also made in other heat wave studies [11]. The RR for the effects of the heat wave was calculated by dividing the number of deaths during the 18-day heat wave period (*A_1_*) by the number of deaths during the 36 reference days (*A*_0_) divided by two:

(1) $$RR=\frac{A_{1}}{A_{0}/2}$$

We calculated 95% confidence intervals (CI) for the RR using a large-sample approach for person-time incidence ratios [38]:

(2) $$95\%CI=e^{\text{ln}RR\pm 1.96\sqrt{1/A_{1}+1/A_{0}}}$$

If the lower limit of the CI was above 1.00, we considered the increase in deaths during the heat wave to be statistically significant. Wald chi-square tests were performed to compare RRs between subcategories of place of death, demographics, causes of death, and building climate zones [38]. Excess deaths, an indication of population burden, were defined as heat wave deaths minus half of reference day deaths:

(3) $$Excess\;deaths=A_{1}-\frac{A_{0}}{2}$$

### 2.4. Definitions of Subgroups and Temperature Data

To examine risks associated with place of death, we calculated RRs for dying inside and outside of a hospital, using death certificate classifications [39]. Out-of-hospital deaths were further analyzed by whether the death occurred in a hospice, nursing or long-term care facility; at home; or elsewhere. 

For demographic, cause-specific, and geographic sub-populations we calculated total (all-place) and at-home heat wave mortality RRs. Demographic variables included sex, 10-year age groups, and race/ethnicity groups. For internal (ICD-10 A00 to R94) and external causes of death (ICD-10 V01 to Y89.9), we used the underlying cause-of-death ICD-10 codes [36]. As in a previous heat wave mortality study, we aggregated specific causes of death into eight categories of internal causes of death (infectious and parasitic, neoplasms, endocrine disease, mental/nervous system, cardiovascular, respiratory system, digestive system, and other internal diseases,) and eight categories of external causes of death (transport accidents, falls, accidental drowning, related to extreme heat, accidental poisoning, suicide, homicide, and other external causes) [30].

To evaluate mortality risk by geographic region across California’s diverse topography, which spans coastal, valley, desert, urban, and mountainous environments, we measured risk across 16 building climate zones. We first excluded deaths of out-of-state residents (1.3%) and then assigned a climate zone to each decedent by geocoding their residential address. The geocoding match rates were 96.1% and 96.4% for deaths that occurred on heat wave days and reference days, respectively. These zones are mandated by California’s Energy Commission [40] and are defined by climate, elevation, and estimated energy consumption (Supplementary Material [SM], Figure S1) [41]. New home construction has been required to meet zone-specific energy-efficiency standards established since 1982 [40]. 

To aid in interpretation of geographic variation, we examined official temperature readings and relative humidity measurements from 1 June to 31 August 2006 throughout California obtained from the California Air Quality and Meteorological Information System [42] and AC ownership data by climate zone as published by Ostro *et al*. 2010, which are results from a California residential survey conducted in 2004 by the California Energy Commission [43]. For the heat wave period and reference days, we followed a formula published by the National Oceanic and Atmospheric Administration to calculate average daily apparent temperatures (ADATs), a heat index that uses relative humidity and ambient temperature to give a measure of human physical comfort as it relates to weather conditions [43]. For each climate zone, “peak” ADAT was calculated to capture the most significant elevation in temperature experienced for a given zone during the 18-day period. This was calculated as an average of ADATs occurring on 22 to 24 July, the three days on which the highest upper-air meteorological measurements occurred [35].

## 3. Results

### 3.1. Place of Death

Overall, there were 582 (5%) more deaths than expected in California during the heat wave period (total mortality RR = 1.05, CI 1.03–1.08; Table 1). Of these excess deaths, 384 (66%) occurred at the decedent’s home. The at-home mortality RR (1.12, CI 1.07–1.16) was significantly greater than that of hospital deaths (*p*-value < 0.01) and hospice or nursing home deaths (*p*-value = 0.01). Furthermore, different demographic, cause of death, and geographical patterns of risk were observed among deaths occurring at home, as described below. 

### 3.2. Demographics 

**Figure 1:**
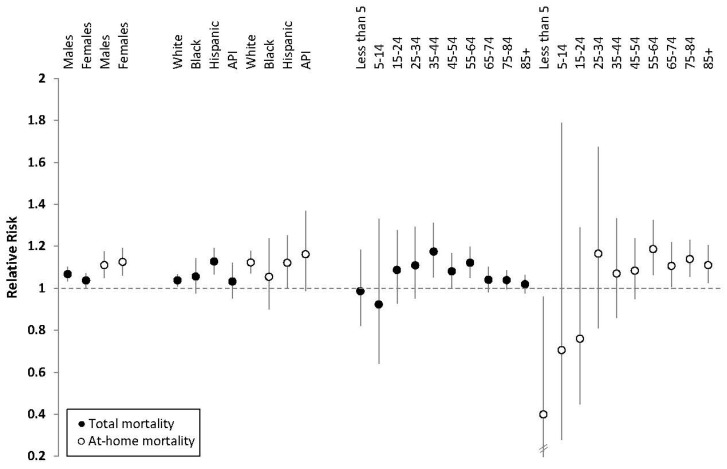
Heat wave relative risks (RRs) for total deaths (solid circles) and deaths that occurred at-home (hollow circles) by demographic group and 95% confidence intervals (CIs). Notes: Vertical lines represent 95% CIs; the CI for at-home deaths among those less than 5 years of age is wider than the RR scale; API is Asian/Hawaiian/Pacific Islander.

Total and at-home RRs for death by sex, race/ethnicity, and 10-year age groups are presented in Figure 1 and Supplementary Material (SM) Table S1. Risk did not significantly differ by sex. Across race/ethnicity, the highest total mortality risk was observed among Hispanics (RR = 1.13, CI 1.07–1.19), followed by blacks, whites, and APIs. The RR for Hispanics was significantly greater than that for whites (RR = 1.04, CI 1.01–1.07, *p*-value < 0.01). By age, total mortality risk was highest among those aged 35–44 (RR = 1.18, CI 1.05–1.31); 45–54 (RR = 1.08, CI 1.00–1.17); and 55–64 (RR = 1.12, CI 1.05–1.20). Total mortality risk estimates among age groups from 35 to 64 were higher than those observed among adults 65 years of age and older. Children in age groups 0–4 and 5–14 appeared to experience null or lower risks than other age groups for total mortality.

Examining deaths occurring at home specifically, mortality risk patterns by race/ethnicity and age varied. While Hispanics experienced higher risk for total mortality than whites, their at-home mortality risk (RR = 1.12, CI 1.00–1.25) did not differ from whites (RR = 1.12, CI 1.07–1.18). Whites’ risk was greater at home than for deaths not occurring at home (*p*-value < 0.001; SM, Table S1). The race/ethnic group with the highest mortality RR point estimate for at-home mortality was APIs (RR = 1.16, CI 0.99–1.37), though the confidence interval was wide and not significantly higher than other race/ethnic groups. By age for at-home deaths, risk was highest among those aged 55–64 (RR = 1.19, CI 1.06–1.32) and elevated across all groups above 65. This is in contrast to mortality not occurring at home, where risk was highest among adults aged 35–44 (SM, Table S1). Among very young children (age 0–4), while total mortality appeared null (RR = 0.99, CI 0.82–1.18), deaths not at home showed a non-statistically significant increase (RR = 1.04, CI 0.86–1.26), and deaths at home showed a statistically significant deficit (RR = 0.40, CI 0.17–0.96). 

### 3.3. Internal and External Causes of Death

Table 2 displays total and at-home mortality RRs during the heat wave by internal and external causes. Total mortality risk due to internal causes increased (RR = 1.04, CI 1.02–1.07). Among specific internal causes, most RR point estimates were elevated, with the highest risk observed among mental/nervous system disorders (RR = 1.11, CI 1.03–1.20). Cardiovascular diseases accounted for the category with the greatest number of excess deaths during the heat wave, 155 (40%) of 408. Total mortality risk due to external causes of death (RR = 1.18, CI 1.10–1.27) was greater than that of internal causes of death, though deaths related to external causes are far fewer. Of the specific external causes, deaths due to extreme heat (RR = 15.23, CI 8.54–27.15; 92 excess deaths) had the greatest risk. Deaths due to accidental poisonings were also significantly increased during the heat wave (RR = 1.26, CI 1.04–1.51). Among the five other specific external causes, all but suicides had RR estimates above 1.00, although most were not statistically significant. 

The cause-of-death risk patterns for at-home mortality differed from deaths not at home. For internal causes of death, at-home mortality risk during the heat wave was elevated (RR = 1.11, CI 1.06–1.16), although mortality elsewhere was not (RR = 1.01, CI 0.98–1.04; *p*-value < 0.001; SM, Table S2). Of the eight specific internal organ/disease categories we examined, at-home mortality was elevated during the heat wave for all categories other than digestive, neoplastic, and infectious/parasitic. Statistically significant increases in mortality risk were observed for mental/behavioral disorders, cardiovascular disease, and endocrine disease. For cardiovascular disease, the mortality risk among at-home deaths (RR = 1.18, CI 1.10–1.26) was greater than mortality risk among those not at home, where an increase in risk was not observed (RR = 0.99, CI 0.94–1.03, *p*-value < 0.001; SM, Table S2). Mortality due to external causes occurring at home (RR = 1.26, CI 1.08–1.47) was slightly elevated compared to not at home (RR = 1.15, CI 1.06–1.25), though not significantly so (*p*-value = 0.33; SM, Table S2). Among at-home deaths from external causes, deaths were significantly elevated in relation to extreme heat (RR = 86.0, CI 11.8–624.5) and accidental drowning (RR = 8.0, CI 1.7–37.8).

### 3.4. Geography

**Figure 2:**
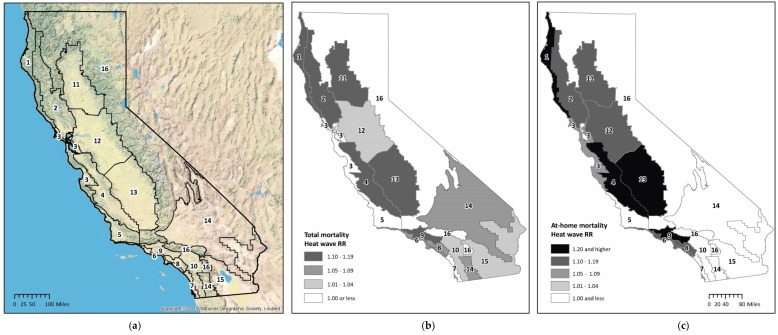
Heat wave total and at-home all-cause mortality relative risks (RRs) for California’s sixteen building climate zones: (**a**) California’s sixteen building climate zones and topography; (**b**) Total mortality heat wave relative risk by building climate zone; (**c**) At-home mortality heat wave relative risk by building climate zone.

Total heat wave mortality was widespread across the state, with most zones showing an increase in the total mortality RR (Table 3, Figure 2). However, magnitude of total mortality risk varied among zones with elevated RRs, especially for deaths at home. The southern portion of California’s Central Valley, a large agricultural basin in the center of the state bordered by the Pacific coastal ridge on the west and the Sierra Nevada mountain ranges on the east, was among the zones with the highest total mortality (Zone 13, RR = 1.11, CI 1.02–1.22). Two other zones with significantly elevated total mortality risk were in the coastal interior (Zones 4 and 9). Zone 9, which includes the interior part of Los Angeles County, is a densely populated, urban environment, and it experienced the largest absolute number of excess deaths (n = 168). The relative risk in the zone encompassing the majority of California’s mountainous, non-coastal areas suggested no apparent increase or a possible decrease (Zone 16, RR = 0.90, CI 0.72–1.11). At-home mortality risks in certain zones were sharply higher: high-risk areas for deaths occurring at home again included the southern Central Valley (Zone 13, RR = 1.43, CI, 1.21–1.68) and the far north coast (Zone 1, RR = 1.58, CI 1.01–2.48; Table 3, Figure 2).

Across all climate zones, temperatures (peak period ADATs) consistently exceeded the reference days’ average through most of the heat wave period (SM, Table S3). The hottest area during the heat wave peak (94 °F ADAT) was a desert area (Zone 15); although this zone did not have a significant increase in mortality, it had the highest percentage of AC ownership, 97%. The highest mortality point estimates were observed in the northernmost coastal climate zone noted above (Zone 1); this zone experienced the largest shift between typical and peak ADAT temperatures (23 °F differential), with the lowest percentage (4%) of homes equipped with AC (Table 3; SM, Table S3). 

## 4. Discussion

Overall, California’s population experienced a 5% increase in total deaths during the heat wave period. Although other mortality studies have investigated the impact of the 2006 heat wave in California, this is the first to examine at-home mortality risk by demographic group, internal and external causes, and building climate zones. The heat wave impact appeared amplified among those who died at home, where a 12% increase was seen, representing 66% of total excess deaths. Further, non-elderly adults and Hispanics experienced sharper increases in mortality risk compared to elderly and whites, respectively. These results differ from demographic findings of some previous studies, as discussed below. While internal causes of death are more commonly investigated in the heat wave mortality literature, external causes of death were significantly elevated during the heat wave period. We also identified differing patterns among those dying at home and geographic variability by climate zone.

The higher at-home *versus* outside the home mortality risk identified in this study coincides with other work that investigated heat-related vulnerabilities at home [11,12,44]. A Canadian study observed increased mortality risk among those at home compared to those inside a health care institution during a summer hot period [11]. Heat wave studies in Chicago and Paris found that living alone was one of the strongest risk factors for death [12,44], while having social contacts nearby was protective [12]. A separate analysis of 140 fatalities investigated by the coroner as related to the California heat wave found that 46 percent lived alone, and of these, 19% died even though a social contact had contacted or visited them within the 24 h prior to death [45]. We did not locate other studies examining risk of death by age, race/ethnicity, and internal or external causes of death among those dying at home, and the differences identified in the present study suggests the need for further evaluation of these patterns for risk at-home compared to other locations.

Our observed demographic patterns differ in some respects from other studies of mortality in the U.S. and California on days of high heat [14,16], and restricting our demographic analysis to internal causes, as other studies have done, did not change our findings (SM, Table S4). However, higher risk from persistent extreme temperatures has also been found among non-elderly adults in Sweden [21], and elevated risk of heat-related fatalities has been noted among U.S. agricultural workers from Central and South America [21,46]. The pattern of increased risk among non-elderly adults seen here may be due, in part, to these persons being unaware of the increased risk, thus continuing with normal activity patterns and not taking protective action. Perceived threat of heat has been observed as a main contributing factor to following heat warnings, and these middle-aged adults may not perceive themselves to be at risk [47].

Differences in heat effects across studies may also be due at least in part to varying definitions of a heat wave and the duration and intensity of the heat event. Estimates of the added health impact of a heat wave have ranged from a relatively small effect (0.2% to 3.8% excess relative risk) that was apparent only after four consecutive heat wave days [48], to an estimate of increased risk of 1.5 to 3.0 times higher during periods of long duration and high intensity [23]. In addition, some effects may have delayed manifestation. For example, in one study, the maximum respiratory mortality occurred six days after the heat wave [49]. Shorter heat events (e.g*.*, two or more days in duration), are considered a heat wave in some analyses [15,25], but the mortality impact could be substantially different than what occurs during a more extended period of high heat. Rocklov *et al*. modeled the effects of heat intensity and duration on mortality to quantify these relationships [22]. They concluded that the additional stress effects from cumulative exposure may be different from those from shorter periods of heat, including potentially different patterns of susceptibility. They observed the additional effect from the more extended heat wave appeared to impact adult and elderly populations more significantly than the very elderly (age 80 and over). This analysis is consistent with our findings of greater heat wave mortality RRs among non-elderly adults. We also noted elevations that were not statistically significant among younger adults (<45 years) not studied by Rocklov, although these findings were not statistically significant. The shift in impact based on the greater duration and intensity accompanying a heat wave may also have implications for younger age categories, as outcomes may be modified by a combination of altered behaviors and physiological stress during these more extreme heat events.

In addition to the finding that young people appeared relatively less affected than adults, among those deaths at home, the significant deficit among the youngest group for at-home deaths, infants to age four, followed by substantial if not statistically significant deficits continuing in other younger age groups, suggests the potential for a protective effect for infants and young persons at home. A recent review by Xu *et al*. of the impact of heat waves on children’s health found inconsistent results for mortality [50]. However, health impacts may occur, but may be more easily evident in morbidity rather than mortality outcomes; e.g*.*, young children age 0–4 were at highest risk for emergency department visits [34]; and specific elevated risks have been noted for renal and respiratory disease, electrolyte imbalance, and fever [50]. Xu *et al*. hypothesized that very young children under one year of age may be most vulnerable, a finding supported by a more recent study focusing on temperature and infant mortality [51]. This is also consistent with our finding of a slightly elevated (non-significant) risk of deaths among children when not at home. The seeming discrepancy with the present study’s findings of low risk in young persons, at least regarding at-home deaths may in part be due to behavioral and care patterns, especially during a clearly recognized extensive period of elevated heat, rather than a lack of underlying biological risk during heat. For example, infants and very young children will likely have the protection of parents who can make decisions for them to stay indoors or take them to the emergency room if needed, whereas the elderly may not receive needed medical attention, or have the ability to recover. This finding of lack of harm in young children may also reflect effectiveness of heat warnings. 

Race/ethnicity differences were apparent in our findings. Although overall at-home mortality was greater than mortality not-at-home, consistent with existing literature, our study identified race/ethnic group differences. Whites and API’s experienced greater risk for at-home *versus*. not-at-home mortality, but this contrast was not apparent among Hispanics, Blacks, or those of other/mixed backgrounds, who had similar risk regardless of setting. Although we have no overarching hypothesis for why the pattern of higher at-home risk failed to hold across all race/ethnic groups, at least in Hispanics, the increase in not-at-home deaths may reflect occupational exposures, which the present study lacked the data to address. In California, Hispanics are often employed in agriculture or construction/labor [52], and this may contribute to the elevated total mortality risk among Hispanics compared to whites [46]. While this study did not find APIs to have higher total mortality than other races, a study of emergency department visits related to ambient heat found varying risk among Asians, with elevated risk for some specific disease outcomes [53]. One study of Medicare claims found lower rates of both in-patient and out-patient visits by Asians for hyperthermia claims [54]. The approximately 4% overall increase in total mortality risk by internal causes of death and the greatest increase for mortality risk due to cardiovascular diseases identified in the current study are similar to findings in other heat wave studies in the U.S., Sweden, and Spain [17,22,30]. For at-home deaths, we found mortality due to cardiovascular disease had elevated risk, as well as the greatest number of excess deaths compared to other causes of death. Although the current analysis does not isolate this risk from other known risk factors associated with home characteristics (e.g*.*, AC use, social isolation, socioeconomic status), this suggests that having effective heat advice for those with preexisting cardiovascular conditions could reduce the number of deaths occurring at home during heat waves. 

Few studies have examined external causes of death during heat waves. Heat wave mortality risk by external causes of death has been examined in one study in Spain, which found patterns similar to our study, including elevated risk for deaths due to extreme heat exposure, accidental drowning, and accidental poisoning [30]. Increased risk of death due to accidental poisoning during heat waves has been demonstrated in one other study in New York City, in which the authors suggested that decreased thermoregulatory capacity of addiction may play a role [55]. In a sub-analysis of our data, we found that this category of accidental poisoning includes deaths from various prescription medications. Certain classes of drugs can reduce the body’s ability to cope with heat, including many commonly used medications such as vasoconstrictors, diuretics, beta blockers, and antidepressants or antipsychotics [56]. Although the number of deaths due to accidental poisonings is fewer than other major causes of death, further characterization of these risks would help in the development of heat advice for certain prescription medication use. The pattern of excess mortality risk for homicides found here, although not statistically significant, has been observed in past studies of heat [31,57]. Literature on weather and suicide has been mixed, suggesting that relationship may vary with specific climatic conditions [31,58,59].

The broad geographic scope of the heat wave resulted in impacts across the state’s varying building climate zones, and most ADATs for the peak period exceeded the threshold at which mortality increases have been noted in U.S. and European cities (65 to 75 °F) [18,19]. Still, significant differences in mortality across zones were evident. While the nature of these data did not allow the rigorous statistical analysis required to separate independent effects, several noteworthy issues emerge. The variability in mortality during the heat wave period between geographic regions is likely mitigated not only by temperature, but also presumably by a complex interplay of AC and other factors. Increased mortality in particular geographic areas may reflect additional underlying local vulnerability factors, such as poor housing conditions, barriers to medical care, high-exposure work conditions, and prevalence of migrant farmworker populations. 

Our geographic findings support the importance of AC as a protective factor, based on the resilience noted in both an area with extremely high heat but nearly comprehensive AC ownership, and in contrast, the impacts in coastal or coastal interior areas with lower AC ownership. This finding is consistent with studies showing populations in cooler climates may be less adapted to increased temperature [26,60]. The earlier referenced coroner’s study of California heat wave fatalities found only one person of those who died indoors was reported to have been using AC prior to death, and a majority either lacked AC or their AC was not functional [45]. A recent review also found increased risk in areas of low AC ownership [8], whereas a Texas heat wave study found no significant mortality impact in a very high AC environment (~98% ownership) [61]. 

However, while air conditioning appears essential to protect public health, growth of air conditioning use is problematic in itself, as it is one of the largest drivers of global energy demand [62], thus contributing to global warming by increasing greenhouse gas emissions. Increasing AC use also poses a significant health equity challenge, as persons of lower socioeconomic status face unequal barriers in using AC or evacuating to cooling centers [47]. To address heat in the built environment, innovations have been suggested such as green or cool roofs [5,63,64]. A study in France found building insulation to be protective against mortality in the 2003 heat wave [65]. Notably, the less risk-affected mountainous zone of California in our study has the strictest energy-efficiency building codes [40], although specific conclusions are beyond the scope of this analysis. Still, our findings along with others suggest that nearly complete AC saturation may be required to prevent mortality. 

### Other Considerations and Limitations

Data on several factors that may modify individual mortality risk during a heat wave were not available in the current analysis, including social and cultural isolation, socioeconomic status/income, occupation, access to air conditioning, building insulation of decedent’s home, and indoor temperatures [13]. Further, a power outage occurred in Los Angeles during the heat wave [37]. Any of these factors could have independently contributed to mortality. Nonetheless, the descriptive patterns of risk observed in our analysis help identify important contributors to mortality during periods of increased heat. Our study was based on one heat wave period, while other studies have included multiple exposure years and reference periods [17,23,24,25]. Although our design may restrict power or generalizability of findings, use of the same summer as reference provides control for unidentified and unmeasurable factors, such as underlying demographics that may change over years.

## 5. Conclusions

Our findings suggest the mortality impact of this statewide heat wave extended broadly, but with varying impacts across subpopulations. Future research should examine the underlying reasons for these differential vulnerabilities, ideally employing designs allowing rigorous investigation of demographic and social/behavioral factors (e.g*.*, detailed age patterns, race/ethnic differences, and occupational risk that may be specific to a group such as Hispanics) and physical environment factors (e.g*.*, AC access). Such studies should attempt to parse how these factors may interact to influence mortality patterns, such as at-home risk. Overall, the results can inform much-needed evaluation studies of interventions and public health guidance. More detailed study of causes of death, including relatively unexamined areas such as accidental poisoning and mental/nervous system deaths, and expanding the analysis to include multiple causes of death in addition to primary cause, could deepen our understanding of anticipated human heat effects.

Results from our study may help inform current local, regional, and global public health endeavors to reduce mortality risk during periods of extended heat [1,5,6]. Our findings of higher mortality risk among certain populations may raise awareness among people who may not recognize their risk, including non-elderly adults and persons with preexisting conditions, such as cardiovascular diseases, substance abuse disorders, and patients using certain classes of medications. Messages aimed at reducing accidental deaths during a heat wave are also warranted. In light of projected continuing increases in the frequency of heat periods, the observed elevations in heat wave related mortality underscore the need to reduce mortality across multiple vulnerable populations and places, in a manner that is both comprehensive and addresses equity. 

## Figures and Tables

**Table 1:** Heat wave mortality relative risks (RR), 95% confidence intervals (CI), and excess deaths by place of death.

Place of Death	Heat Wave Deaths ^a^	Reference Day Deaths/2 ^b^	RR (CI)	Excess Deaths ^c^
All deaths	11,727	11,144	1.05 (1.03–1.08)	582
Inside hospital ^d^	4933	4820	1.02 (0.99–1.06)	113
Outside hospital	6670	6197	1.08 (1.05–1.11)	473
Decedent‘s home	3646	3262	1.12 (1.07–1.16)	384
Hospice or nursing home	2218	2156	1.03 (0.98–1.08)	62
Other	806	779	1.04 (0.95–1.13)	27
Unknown	124	127	0.97 (0.79–1.21)	−3

Notes: ^a^ Deaths that occurred between 15 July to 1 August, 2006. ^b^ Deaths that occurred on same-summer reference days divided by two and then rounded for presentation. ^c^ Heat wave deaths minus reference day deaths. ^d^ Includes in-patient, emergency room/outpatient, and dead-on-arrival deaths.

**Table 2:** Heat wave mortality relative risk and excess deaths for all deaths and deaths that occurred at-home, by underlying cause of death.

Underlying Cause of Death (ICD-10 Codes)	Total Mortality				At-Home Mortality			
	Heat Wave ^a^	Reference/2 ^b^	Relative Risk (CI)	Excess Deaths	Heat Wave	Reference/2	Relative Risk (CI)	Excess Deaths
Internal causes (A00–R94)	10,575	10,167	1.04 (1.02–1.07) *	408	3382	3053	1.11 (1.06–1.16) *	330
Mental/nervous system (F00–H95)	935	841	1.11 (1.03–1.20) *	94	259	212	1.22 (1.05–1.43) *	47
Endocrine Disease (E00–88)	483	448	1.08 (0.97–1.20)	35	163	119	1.38 (1.13–1.68) *	45
Respiratory system (J00–99)	1042	973	1.07 (0.99–1.16)	69	243	217	1.12 (0.95–1.31)	26
Other internal diseases (L00–R99)	515	488	1.06 (0.95–1.17)	27	88	77	1.14 (0.87–1.48)	11
Cardiovascular (I00–99)	4169	4014	1.04 (1.00–1.08)	155	1292	1095	1.18 (1.10–1.26) *	198
Digestive system (K00–92)	443	434	1.02 (0.91–1.14)	9	67	71	0.94 (0.70–1.25)	−5
Neoplasms (C00–D48)	2769	2739	1.01 (0.97–1.06)	30	1249	1231	1.01 (0.95–1.09)	18
Infectious and parasitic (A00–B99)	219	230	0.95 (0.81–1.12)	−11	21	29	0.71 (0.43–1.17)	−9
External causes (V01–Y89.9)	1152	977	1.18 (1.10–1.27) *	175	264	210	1.26 (1.08–1.47) *	54
External causes, excluding X30	1053	971	1.09 (1.01–1.17) *	83	221	209	1.05 (0.90–1.24)	12
Related to extreme heat (X30)	99	7	15.2 (8.54–27.1) *	92	43	1	86.0 (11.8–624.5) *	43
Accidental drowning (W65–74)	57	43	1.33 (0.95–1.85)	14	8	1	8.00 (1.70–37.67) *	7
Accidental poisoning (X40–49)	182	145	1.26 (1.04–1.51) *	37	88	69	1.28 (0.98–1.67)	19
Homicide (X85–Y09, Y87.1)	170	145	1.17 (0.97–1.42)	25	16	18	0.89 (0.49–1.60)	−2
Falls (W00–19)	91	83	1.09 (0.84–1.41)	8	5	7	0.67 (0.24–1.83)	−3
Transport accidents (V01–99, Y85)	292	278	1.05 (0.91–1.21)	14	1	1	0.67 (0.07–6.41)	−1
Other external ^c^	92	91	1.01 (0.79–1.30)	1	12	16	0.75 (0.39–1.46)	−4
Suicide (X60–84, Y87.0)	169	185	0.91 (0.76–1.10)	−16	91	97	0.94 (0.73–1.21)	−6

Notes: * indicates lower bound of 95% CI is above 1.00. ^a^ Deaths that occurred between 15 July to 1 August, 2006. ^b^ Deaths that occurred on same-summer reference days divided by two and then rounded for presentation. ^c^ Other external cases include ICD-10 codes for other/unspecified accidents, and causes of undetermined intent/legal intervention, or medical/surgical complications (Y10–Y35, Y40–Y84, Y87.2, Y88, Y89.0, and Y89.9).

**Table 3:** Climate zone characteristics, heat wave mortality relative risks and excess deaths for all deaths and deaths that occurred at-home, temperature during the heat wave, and AC ownership.

Climate Zone	Representative City/Climate Type ^a^	Total Mortality		At-Home Mortality		ADAT during Peak Heat Wave ^b^ °F (°C)	Peak Heat Wave ADAT Differential (°F) ^c^	% AC Ownership ^d^
		Relative Risk (CI)	Excess Deaths	Relative Risk (CI)	Excess Deaths			
1	Arcata/coastal	1.19 (0.89–1.59)	12	1.58 (1.01–2.48) *	13	81 (27)	23	4%
4	Sunnyvale/coastal interior	1.14 (1.02–1.26) *	65	1.22 (1.01–1.46) *	32	82 (28)	17	48%
2	Santa Rosa/coastal interior	1.14 (1.00–1.29)	45	1.10 (0.89–1.36)	13	85 (29)	10	43%
11	Red Bluff/N Central Valley	1.12 (1.00–1.26)	50	1.18 (0.97–1.44)	24	87 (31)	14	89%
13	Fresno/S Central Valley	1.11 (1.02–1.22) *	74	1.43 (1.21–1.68) *	75	88 (31)	13	89%
9	Pasadena/coastal interior	1.10 (1.04–1.17) *	168	1.20 (1.08–1.34) *	86	84 (29)	15	76%
n/a ^e^	n/a	1.10 (0.98–1.23)	42	1.32 (0.92–1.91)	12	n/a	n/a	n/a
6	Los Angeles/coastal	1.06 (0.98–1.15)	51	1.10 (0.95–1.27)	26	74 (23)	11	31%
8	El Toro/coastal	1.05 (0.98–1.13)	61	1.19 (1.05–1.36) *	58	79 (26)	11	55%
14	China Lake/desert	1.05 (0.91–1.21)	14	0.98 (0.77–1.24)	−2	85 (29)	10	88%
10	Riverside/coastal interior	1.03 (0.96–1.11)	39	0.97 (0.86–1.10)	-11	84 (29)	13	87%
12	Sacramento/Central Valley	1.02 (0.96–1.08)	27	1.11 (0.99–1.25)	48	88 (31)	19	86%
15	El Centro/desert	1.02 (0.87–1.20)	6	0.98 (0.75–1.29)	−2	94 (34)	8	97%
3	Oakland/coastal	0.99 (0.92–1.06)	−11	1.06 (0.93–1.21)	21	72 (22)	14	11%
7	San Diego/coastal	0.97 (0.88–1.07)	−20	0.97 (0.81–1.15)	−7	78 (26)	12	32%
5	Santa Maria/coastal	0.90 (0.72–1.11)	−14	0.98 (0.68–1.40)	−1	76 (24)	15	15%
16	Mount Shasta/mountainous	0.90 (0.76–1.06)	−24	1.00 (0.76–1.31)	0	78 (26)	13	56%

Notes: ADAT is average daily apparent temperature; * indicates lower bound of 95% CI is above 1.00. ^a^ Representative city and climate type as defined by the California Energy Commission; city is not necessarily the zone’s geographic center (CEC, 1981). ^b^ The peak of the heat wave was 22 to 24 July 2006. ^c^ ADAT during peak of the heat wave minus ADAT on reference days. ^d^ From Ostro *et al*., 2010. ^e^ Includes deaths to those living out of California (1.3% of all deaths), and the deaths for which residential address was incomplete and could not be geocoded (3.7% of all deaths).

## Supplementary Materials

The following are available online at www.mdpi.com/1660-4601/13/3/299/s1, Figure S1: California counties and sixteen building climate zones; Table S1: Total, at-home, and non-at-home mortality by demographic variables (sex, race/ethnicity, 10-year age groups): Heat wave and reference day death counts, relative risks, 95% confidence intervals (CI), and excess deaths; Table S2: Non-at-home mortality by internal and external causes of death: Heat wave and reference day death counts, relative risks, confidence intervals, and excess deaths; Table S3: Average daily apparent temperature (ADAT) during heat wave (HW) and reference days by building climate zone; and Table S4: Mortality restricted to internal causes by demographic variables (sex, race/ethnicity, 10-year age groups): Heat wave and reference day death counts, relative risks, confidence intervals, and excess deaths.

## Abbreviations

The following abbreviations are used in this manuscript:

AC	Air conditioning
API	Asian/Hawaiian/Pacific Islanders
CI	Confidence interval
RR	Relative risk
