# Supplementary material for: Mortality during a Large-Scale Heat Wave by Place, Demographic Group, Internal and External Causes of Death, and Building Climate Zone
Source: Int J Environ Res Public Health. 2016 Mar 9;13(3):299. doi: 10.3390/ijerph13030299 (PMC4808962; doi:10.3390/ijerph13030299)
Supplement: Supplementary file 1 [file ijerph-13-00299-s001.pdf]

Supplementary Materials: Mortality during a Large-Scale Heat Wave by Place, Demographic Group, Internal and External Causes of Death, and Building Climate Zone

Lauren Joe, Sumi Hoshiko, Dina Dobraca, Rebecca Jackson, Svetlana Smorodinsky, Daniel Smith and Martha Harnly

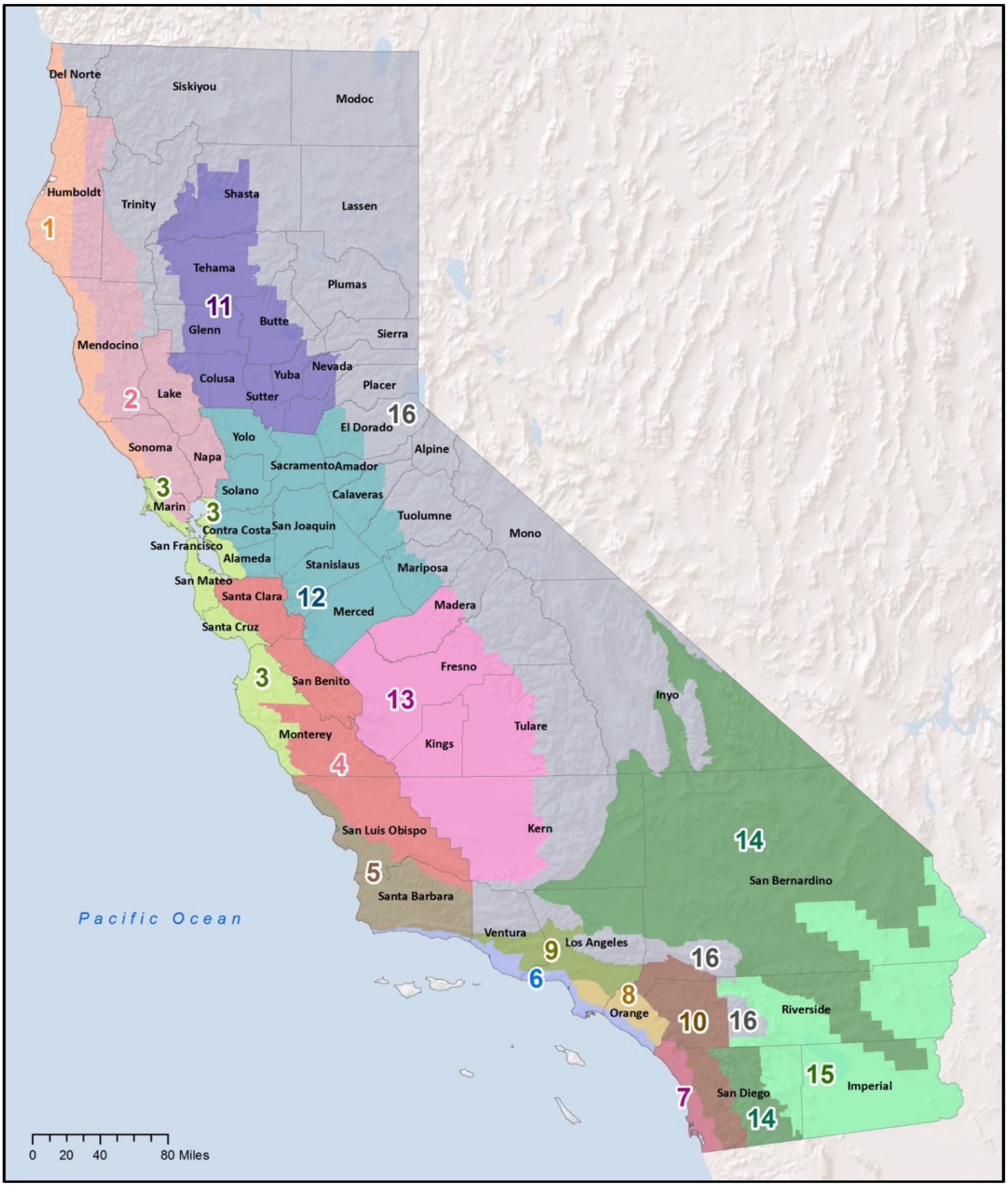

Figure S1. California counties and sixteen building climate zones *. Note: * Boundaries from: California Energy Commission (CEC), California Energy Maps—California Building Climate Zone Areas [1].

Table S1. Total, at-home, and non-at-home [a] mortality by demographic variables (sex, race/ethnicity, 10-year age groups): Heat wave and reference day death counts, relative risks, 95% confidence intervals (CI), and excess deaths.

	Total Mortality				At-Home Mortality				Non-at-Home Mortality			
	Heat Wave [b]	Reference/2 [c]	Relative Risk (CI)	Excess Deaths [d]	Heat Wave	Reference/2	Relative Risk (CI)	Excess Deaths	Heat Wave	Reference/2	Relative Risk (CI)	Excess Deaths
**Sex**												
Males	6080	5698	1.07 (1.03–1.10)	382	1878	1692	1.11 (1.05–1.17)	186	4119	3933	1.05 (1.01–1.09)	186
Females	5647	5446	1.04 (1.00–1.07)	201	1768	1571	1.13 (1.06–1.19)	197	3838	3821	1.00 (0.97–1.04)	16
**Race/Ethnicity**												
White	7876	7586	1.04 (1.01–1.07)	290	2667	2374	1.12 (1.07–1.18)	293	5120	5119	1.00 (0.97–1.03)	1.5
Black	948	897	1.06 (0.98–1.14)	51	230	218	1.06 (0.90–1.24)	12	704	669	1.05 (0.96–1.15)	35
Hispanic	1908	1691	1.13 (1.07–1.19)	217	488	436	1.12 (1.00–1.25)	52	1407	1241	1.13 (1.06–1.21)	166
Asian, Hawaiian/PI	875	848	1.03 (0.95–1.12)	27	226	195	1.16 (0.99–1.37)	31	643	648	0.99 (0.90–1.09)	−5
Other, Multi, AIAN [e]	120	122	0.98 (0.79–1.22)	−2	35	40	0.86 (0.58–1.28)	−5	84	78	1.06 (0.81–1.38)	5
**Age**												
Less than 5	171	174	0.99 (0.82–1.18)	−3	6	15	0.40 (0.17–0.96)	−9	164	157	1.04 (0.86–1.26)	7
5–14	42	46	0.92 (0.64–1.33)	−4	6	9	0.71 (0.28–1.79)	−3	34	36	0.94 (0.63–1.42)	−2
15–24	232	214	1.09 (0.93–1.28)	18	19	25	0.76 (0.45–1.29)	−6	203	183	1.11 (0.93–1.32)	20
25–34	254	229	1.11 (0.95–1.29)	25	46	39	1.16 (0.81–1.68)	7	204	183	1.11 (0.94–1.32)	21
35–44	503	428	1.18 (1.05–1.31)	75	122	114	1.07 (0.86–1.33)	8	373	307	1.21 (1.07–1.38)	66
45–54	1011	936	1.08 (1.00–1.17)	75	335	309	1.08 (0.95–1.24)	26	664	613	1.08 (0.98–1.19)	51
55–64	1423	1269	1.12 (1.05–1.20)	154	514	433	1.19 (1.06–1.32)	81	894	821	1.09 (1.00–1.18)	73
65–74	1794	1725	1.04 (0.98–1.10)	69	643	581	1.11 (1.01–1.22)	62	1133	1119	1.01 (0.94–1.09)	14
75–84	3064	2949	1.04 (0.99–1.09)	115	1039	913	1.14 (1.06–1.23)	126	1992	2004	0.99 (0.94–1.05)	−13
85+	3233	3174	1.02 (0.98–1.06)	59	916	825	1.11 (1.02–1.20)	91	2296	2327	0.99 (0.94–1.04)	−31

Notes: [a] There were 7957 heat wave deaths that did not occur at home. Non-at-home deaths included deaths that occurred inside a hospital, at hospice or nursing, or other place outside of a hospital. Deaths that occurred at unknown places were not included; [b] Deaths that occurred between 15 July to 1 August 2006; [c] Deaths that occurred on same-summer reference days divided by two and then rounded for presentation; [d] Heat wave deaths minus reference day deaths; [e] Includes other, multi-race, and American Indian/Alaska Native race/ethnicities.

Table S2. Non-at-home [a] mortality by internal and external causes of death: Heat wave and reference day death counts, relative risks, confidence intervals, and excess deaths.

Underlying Cause of Death (ICD-10 Codes)	Non-at-Home Mortality			
	Heat Wave [a]	Reference/2 [b]	Relative Risk (CI)	Excess Deaths [c]
**Internal Causes (A00–R94)**	7106	7016	1.01 (0.98–1.04)	90
Mental/nervous system (F00–H95)	672	622	1.08 (0.98–1.19)	50
Endocrine Disease (E00–88)	318	327	0.97 (0.85–1.11)	−9
Respiratory system (J00–99)	790	746	1.06 (0.97–1.15)	44
Other internal diseases (L00–R99)	419	400	1.05 (0.93–1.18)	19
Cardiovascular (I00–99)	2839	2880	0.99 (0.94–1.03)	−41
Digestive system (K00–92)	371	358	1.04 (0.92–1.18)	13
Neoplasms (C00–D48)	1501	1486	1.01 (0.95–1.07)	15
Infectious and parasitic (A00–B99)	196	197	0.99 (0.84–1.18)	−1
**External Causes (V01–Y89.9)**	851	738	1.15 (1.06–1.25)	113
**External Causes, Excluding X30**	797	732	1.09 (1.00–1.19)	65
Related to extreme heat (X30)	54	6	9.00 (4.81–16.82)	48
Accidental drowning (W65–74)	47	41	1.16 (0.81–1.66)	6
Accidental poisoning (X40–49)	94	76	1.25 (0.96–1.61)	18
Homicide (X85–Y09, Y87.1)	151	124	1.21 (0.99–1.48)	27
Falls (W00–19)	84	74	1.14 (0.87–1.49)	10
Transport accidents (V01–99, Y85)	269	260	1.03 (0.89–1.2)	9
Other external [d]	77	73	1.05 (0.8–1.39)	4
Suicide (X60–84, Y87.0)	75	85	0.88 (0.67–1.16)	−10

Notes: [a] There were 7957 heat wave deaths that did not occur at home. Non-at-home deaths included deaths that occurred inside a hospital, at hospice or nursing, or other place outside of a hospital. Deaths that occurred at unknown places were not included; [b] Deaths that occurred between 15 July to 1 August 2006; [c] Deaths that occurred on same-summer reference days divided by two and then rounded for presentation; [d] Other external cases include ICD-10 codes for other/unspecified accidents, and causes of undetermined intent/legal intervention, or medical/surgical complications (Y10–Y35, Y40–Y84, Y87.2, Y88, Y89.0, Y89.9).

Table S3. Average daily apparent temperature (ADAT) during heat wave (HW) [a] and reference days [b] by building climate zone.

Building Climate Zone	Number of Weather Stations	HW ADAT (°F)	Peak HW ADAT (°F) [c]	Reference Days ADAT (°F)	ADAT Differential (°F) [d]	Peak HW ADAT Differential (°F) [e]	HW Duration (Days) [f]
1	6	69	81	58	11	23	14
2	34	72	85	64	8	10	16
3	32	65	72	58	7	14	16
4	29	74	82	65	9	17	16
5	24	70	76	62	8	15	16
6	24	69	74	62	7	11	18
7	21	73	78	66	7	12	18
8	11	75	79	68	7	11	18
9	27	76	84	69	7	15	16
10	26	77	84	71	6	13	15
11	38	81	87	73	3	14	16
12	65	78	88	69	9	19	14
13	55	82	88	75	7	13	16
14	47	80	85	75	5	10	14
15	32	89	94	86	3	8	15
16	161	73	78	65	8	13	15

Notes: [a] 15 July to 1 August 2006; [b] The same-summer reference days were chosen from the rest of the summer (1 June to 31 August 2006), excluding a holiday period (1–5 July) and the week directly after the heat wave period. Reference days were matched by day-of-the-week at a ratio of two reference days to each heat wave day; [c] During peak of heat wave, 22–24 July 2006; [d] ADAT during the heat wave minus ADAT on reference days; [e] ADAT during peak of the heat wave minus ADAT on reference days; [f] Number of consecutive days including and following 15 July 2006 when ADAT > ADAT on reference days.

Table S4. Mortality restricted to internal causes [a] by demographic variables (sex, race/ethnicity, 10-year age groups) for all places of death: Heat wave [b] and reference day [c] death counts, relative risks (RR), 95% confidence intervals (CI), and excess deaths [d]. (n = 10,575 deaths due to internal causes during heat wave [a]).

	Heat Wave [b]	Reference/2 [c]	Relative Risk (CI)	Excess Deaths [d]
**Sex**				
Males	5258	4992	1.05 (1.02, 1.09)	266
Females	5317	5175	1.03 (0.99, 1.06)	142
**Race/Ethnicity**				
White	7294	7062	1.03 (1.00, 1.06)	232
Black	811	800	1.01 (0.93, 1.10)	11
Hispanic	1558	1417	1.10 (1.03, 1.17)	141
Asian, Hawaiian/PI	805	785	1.03 (0.94, 1.12)	20
Other, Multi, AIAN [e]	107	103	1.03 (0.82, 1.31)	4
**10-Year Age Groups**				
Less than 5	141	149	0.95 (0.77, 1.16)	−8
5–14	27	21	1.29 (0.79, 2.09)	6
15–24	41	43	0.95 (0.66, 1.38)	−2
25–34	110	92	1.19 (0.94, 1.51)	18
35–44	304	283	1.08 (0.94, 1.24)	21
45–54	802	743	1.08 (0.99, 1.18)	59
55–64	1290	1168	1.10 (1.03, 1.18)	122
65–74	1711	1675	1.02 (0.96, 1.08)	36
75–84	2982	2876	1.04 (0.99, 1.08)	106
85+	3167	3117	1.02 (0.97, 1.06)	50

Notes: [a] There were 10,575 deaths due to internal causes during the heat wave. Internal causes of death are ICD-10 codes A00 to R94, which include mental/nervous system, endocrine disease, respiratory system, cardiovascular, digestive system, neoplasm, infection and parasitic, and other internal causes; [b] Deaths that occurred between 15 July to 1 August 2006; [c] Deaths that occurred on same-summer reference days divided by two and then rounded for presentation; [d] Heat wave deaths minus reference day deaths; [e] Includes other, multi-race, and American Indian/Alaska Native race/ethnicities.

References

1. California Energy Maps. Available online: http://www.energy.ca.gov/maps/renewable/building_climate_zones.html (accessed 30 January 2013).

© 2016 by the authors; licensee MDPI, Basel, Switzerland. This article is an open access article distributed under the terms and conditions of the Creative Commons by Attribution (CC-BY) license (http://creativecommons.org/licenses/by/4.0/).
